# Laterality of Ovulation and Presence of the Embryo Do Not Affect Uterine Horn Blood Flow During the First Month of Gestation in Llamas

**DOI:** 10.3389/fvets.2020.598117

**Published:** 2020-12-01

**Authors:** Marcelo H. Ratto, Felipe Urra, Mauricio Silva

**Affiliations:** ^1^Faculty of Veterinary Sciences, Universidad Austral de Chile, Valdivia, Chile; ^2^School of Graduate Studies, Universidad Austral de Chile, Valdivia, Chile; ^3^Department of Veterinary Medicine and Public Health of Veterinary, Universidad Católica de Temuco, Temuco, Chile; ^4^Núcleo de Investigación en Producción Alimentaria, Universidad Católica de Temuco, Temuco, Chile

**Keywords:** llamas, ovulation, embryo, gestation, uterine vascularization

## Abstract

We determined if laterality of ovulation and intrauterine embryo location differentially induces changes in the mesometrial/endometrial vascularization area (MEVA) between uterine horns, during and after embryo migration, elongation and implantation in llamas. Adult, non-pregnant and non-lactating llamas (*n* = 30) were subjected to daily B-mode ultrasound scanning of their ovaries. Llamas with a growing follicle ≥8 mm in diameter in the left (*n* = 15) or right (*n* = 15) ovary were assigned to a single mating with an adult fertile or vasectomized male. Power-doppler ultrasonography was used to determine the MEVA in a cross section of the middle segment of both uterine horns. MEVA was determined by off-line measurements using the ImageJ software. MEVA measurements were performed before mating (day 0) and on days 5, 10, 15, 20, 25, and 30 after mating in pregnant [llamas with left- (*n* = 6) or right-sided (*n* = 6) ovulations] and non-pregnant [llamas with left- (*n* = 6) or right-sided (*n* = 6) ovulations] females. Ovulation was confirmed by the disappearance of a follicle (≥8 mm) detected previously. Pregnancy was confirmed by the presence of the embryo proper. MEVA was analyzed by one-way ANOVA for repeated measures using the MIXED Procedure in SAS. If significant (*P* ≤ 0.05) main effects or interactions were detected, Tukey's *post-hoc* test for multiple comparisons was used. Ovulation rate did not differ (*P* = 0.4) between females mated to an intact or vasectomized male and between right- or left-sided ovulations. Three females mated to the intact and 3 to the vasectomized male did not ovulate and were excluded of the study. First observation of fluid inside the gestational sac and of embryo proper, were made exclusively in the left uterine horn, on day 15.8 ± 3.8 and 22 ± 2.7, and 16.7± 2.6 and 27.5 ± 2.8 for pregnant llamas ovulating in the right and left ovary, respectively. Although the MEVA of both uterine horns was affected by time (*P* < 0.05), it was not affected by physiological status (pregnant vs. non-pregnant; *P* = 0.9) or laterality of ovulation (*P* = 0.4). Contrary to expectations, regardless of the laterality of ovulation, in pregnant llamas the left horn did not display a greater MEVA before or after embryo arrival, a trend that was observed during the first 30 days of gestation.

## Introduction

Llamas and alpacas have several unique reproductive characteristics, one of which is the establishment of embryo implantation and gestation exclusively in the left uterine horn, regardless of laterality of ovulation (1–3). Females from both species have a bicornate uterus that presents a clear asymmetry between uterine horns, with the left horn being larger than its right counterpart (4, 5). This asymmetry is not only observed in pluriparous and pregnant females but also in nulliparous and even in female fetuses, therefore it is not induced by pregnancy (5). Also, the arterial irrigation and venous drainage differ between both uterine horns in llamas. The presence of a prominent cross-over arterial branch extending from the right uterine artery to the left horn suggests that this is irrigated with a greater blood flow (4).

Besides, llamas, and alpacas present a peculiar pattern of intrauterine embryo migration. Although ovulation occurs with the same frequency in the left and right ovary (2, 6), embryos originated from right-ovary ovulations must migrate into the left uterine horn before the day of the beginning of luteolysis (Day 9 after ovulation) for the pregnancy to be successfully established (3, 7).

In most mammalian species significant changes in uterine vascular irrigation are observed during gestation, which are required to initially sustain embryo implantation (8) and latter fetal supply of nutrients and oxygen (9). This phenomenon has been mostly studied in females presenting a symmetric uterus, such as cows (10, 11), buffalo (12) sheep (13–15), goats (15), and mares (16–20), but only few studies have evaluated uterine vascularization during the first month of gestation (11, 16, 17, 20).

In horses and cattle (10, 20) the establishment of pregnancy gradually increases uterine blood flow in close relationship with embryo/fetal growth during gestation. These hemodynamic changes begin before embryo implantation occurs (11, 16, 17) and exponentially increase thereafter (10). Interestingly, the increase in uterine blood flow begins before an intimate contact between the embryo and the endometrium is established (17), and is closely influenced by embryo location (11, 16, 17). Embryo location induces significant differences in blood flow between both uterine horns in cows (10) and mares (17, 20), generating an asymmetrical blood flow in the former and a symmetrical blood provision in the latter before embryo fixation/implantation as a consequence of different intrauterine embryo migration patterns.

As mentioned before, more than 98% of gestations in llamas are carried out in the left uterine horn, therefore embryos originated from right ovulations must migrate to the left horn in order to achieve a successful pregnancy. The striking features of embryo migration and the special uterine vascular arrangement make this species an interesting model to study uterine vascular perfusion and pregnancy development. Therefore, the goal of this study was to determine if intrauterine embryo location differentially induces changes in mesometrial/endometrial vascularization (MEVA) between the right and left uterine horn, during embryo migration, elongation and implantation in llamas. Since an adequate endometrial blood supply is essential for a successful embryo implantation and survival (8, 21, 22), studies on the spatial relationship between the location of the early embryo/conceptus and the degree of uterine vascular perfusion in llamas may shed some light into the mechanisms controlling embryo implantation in the left uterine horn.

## Materials and Methods

The present study was conducted during the breeding season (November–January) at the Universidad Católica de Temuco, Temuco, Chile (38° 45′S−72° 40′W and 122 m above sea level). All procedures were reviewed and approved by the University Bioethics Committee and were performed in accordance with the animal care protocols established by the same institution.

### Animals

Adult non-pregnant, non-lactating llamas [*n* = 30; age: 5–8 y; weight: 120.5 ± 14.1 Kg; mean Body Condition Score: 3.5 out of 5 (range: 3.0–4.0); parity: 3 ± 2] were maintained on pasture supplemented with hay and water *ad libitum*. Llamas were housed indoors at night and offered 250 g/animal of a commercial diet supplement containing 140 g/kg crude protein and 150 g/kg crude fiber. Also, one intact fertile and one vasectomized adult male (ages: 3 and 5 y; weight: 147.5 ± 8.1 Kg; Body Condition Score: 4 and 5, respectively) were kept under similar conditions as the females, but separate at all times from the female herd. Male-female contact was only allowed during the supervised matings. Vasectomy was performed by a standard surgical procedure 1 year before the start of the present experiment in the context of a previous study.

### Experimental Design

Females were examined once daily by transrectal ultrasonography to monitor follicular growth and then by simple randomization were assigned to the following treatment groups: (a) presence of a growing follicle ≥8 mm in diameter in the right ovary and mating with an intact male (*n* = 8), (b) presence of a growing follicle ≥8 mm in diameter in the left ovary and mating with an intact male (*n* = 7), (c) presence of a growing follicle ≥8 mm in diameter in the right ovary and mating with a vasectomized male (*n* = 8), or (d) presence of a growing follicle ≥8 mm in diameter in the left ovary and mating with a vasectomized male (*n* = 7). Mating was validated only if the receptive female adopted the prone position soon after contact with the male and if intromission and copula lasted more than 5 min. After mating, females were examined using B-mode transrectal ultrasonography every 12 h until ovulation or 48 h, whichever came first. Ovulation was confirmed by the sudden disappearance of a follicle (≥8 mm) detected during previous examinations and only ovulated females were incorporated for the transrectal Power-doppler ultrasound examination.

### Power-Doppler Ultrasonographic Evaluation

The area of mesometrial/endometrial vascularization of both uterine horns was evaluated by Power-doppler ultrasonography in all ovulated females using a 5.0 MHz lineal array transducer coupled to a ultrasound monitor (Sonosite M-Turbo, USA) before mating (Day 0 = Day of mating) and on days 5, 10, 15, 20, 25, and 30 between 08:00 a.m. and 12:00 p.m. as described previously (11, 16, 17). In brief, the transducer was placed over a cross section of the middle segment of each uterine horn where a 10 s video-clip was registered. The area of mesometrial/endometrial vascularization was objectively assessed by off-line measurements of the number of colored pixels as an indicator of blood flow area. Three still images of each horn were selected by a blind procedure, and then used for the determination of the number of colored pixels, and the average was used for the statistical analyses. Power Doppler images were selected based on two criteria: (a) proper cross section of the uterine horn and, (b) absence or minimal presence of Power-doppler noise interference. Then, images were recorded, edited, and analyzed using the ImageJ software (NIH open access, USA). A female was considered pregnant when the gestational sac and the embryo proper were detected by ultrasonography.

### Statistical Analyses

Statistical analyses were performed using the Statistical Analysis System software package SAS Learning Edition, version 4.1 (SAS Institute Inc., Cary, NC, USA, 2006). Serial data were compared by analysis of variance for repeated measures (Proc-mixed procedure) to determine the effects of female physiological status (pregnant vs. non-pregnant), laterality of ovulation (right or left ovary), time and treatments-by time interaction on left and right uterine horn MEVA. If significant (*P* ≤ 0.05) main effects or interactions were detected, Tukey's *post-hoc* test for multiple comparisons was used to locate differences. All data are reported as mean ± SEM, and probabilities ≤ 0.05 were considered significant.

## Results

**Figure 1:**
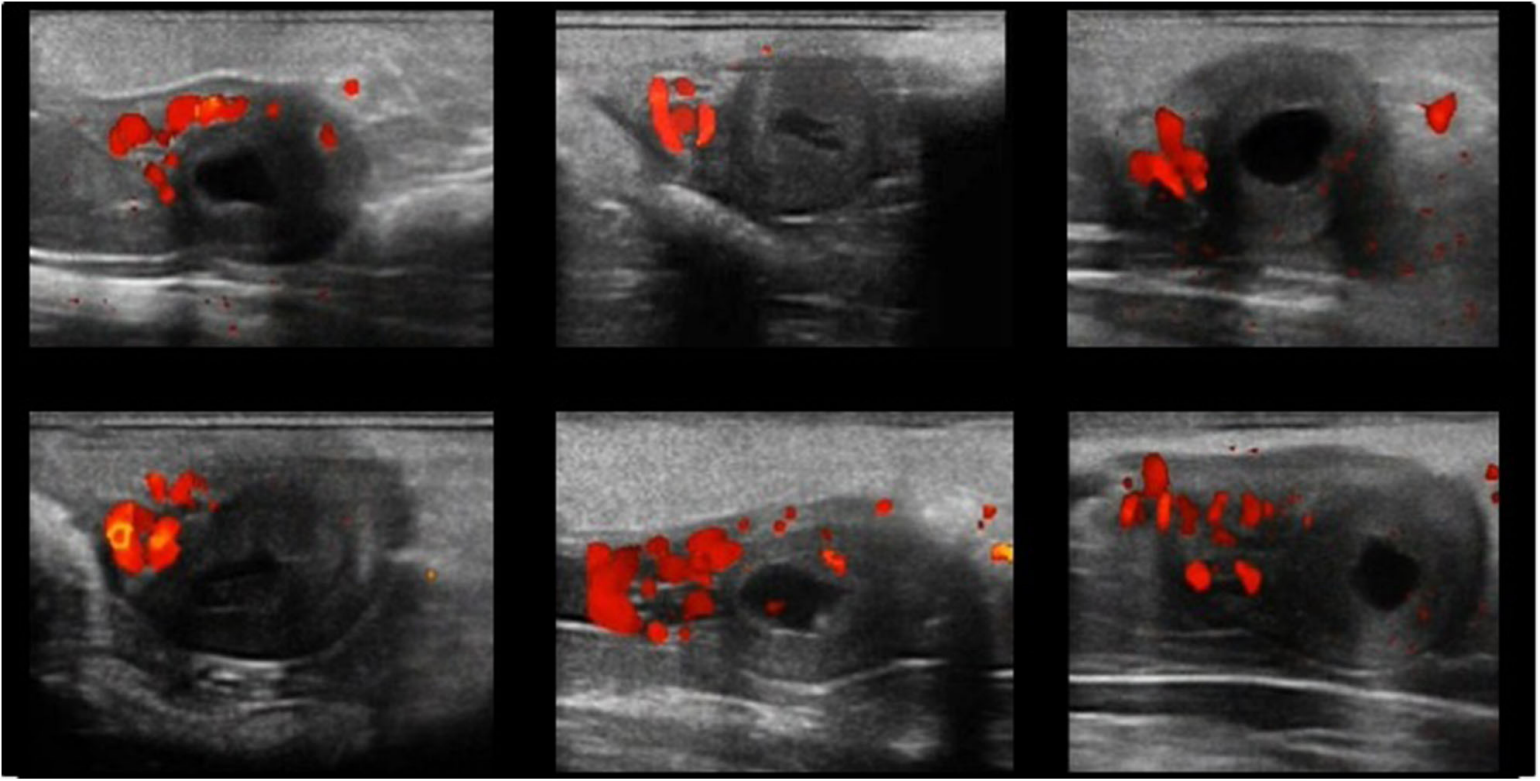
Representative Power-doppler ultrasound images of mesometrial/endometrial vascularization area in gravid uterus (left uterine horn) 30 days after mating in llamas. Each image represents a different female.

There was not a significant difference (*P* = 0.4) in ovulation rate between llamas mated with an intact fertile or vasectomized male. Six out of 8 and 6/7 llamas with a preovulatory follicle ≥8 mm diameter located in either the right or left ovary ovulated and became pregnant after mating with the intact fertile male. Similarly, 6/8 and 6/7 llamas with a preovulatory follicle ≥8 mm diameter located either in the right or left ovary ovulated after mating with the vasectomized male. In pregnant females the earliest ultrasound signs of gestations were observed exclusively in the middle section of the left uterine horn. First observations of fluid inside the gestational sac (i.e., embryonic vesicle) and the embryo proper were recorded on day 15.8 ± 3.8 and 22 ± 2.7, and 16.7± 2.6 and 27.5 ± 2.8, for pregnant llamas ovulating in the right and left ovary, respectively. Representative images of MEVA in gravid uterus (i.e., left uterine horn) 30 days after mating for 6 different females are shown in Figure 1.

**Figure 2:**
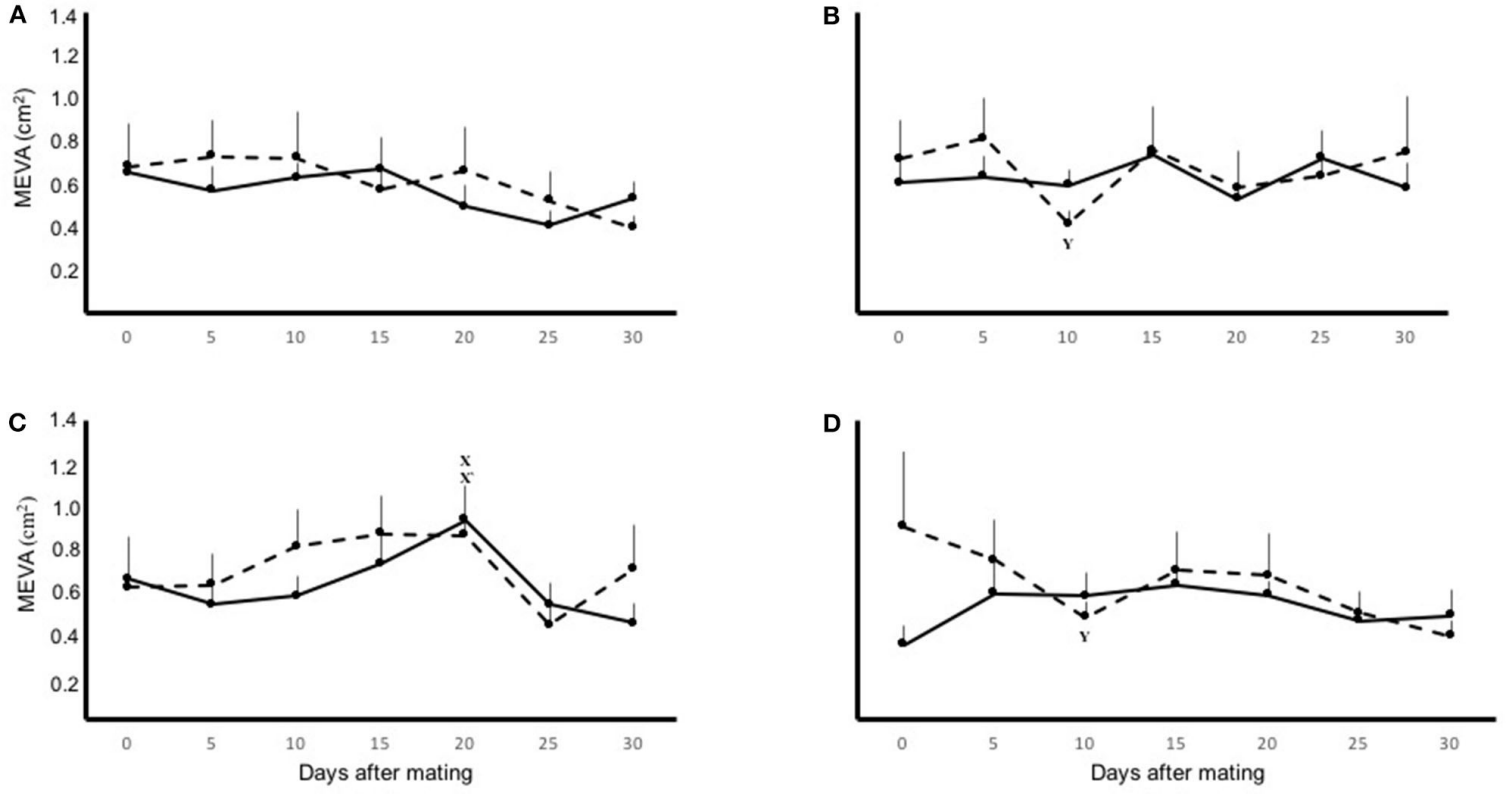
Mean ± SEM mesometrial/endometrial vascularization area (MEVA) on the left (solid line) and right (dashed line) uterine horn, in pregnant **(A,B)** and non-pregnant **(C,D)** llamas with right- **(A,C)** or left-sided **(B,D)** ovulations, during the 30 day period of evaluation. Effect of: physiological status (*P* = 0.9); laterality of ovulation (*P* = 0.4); time (*P* < 0.05). ^**x, x**^′Within uterine horn, the first significant increase from basal MEVA (*P* < 0.01). ^**y**^Within uterine horn, the first significant decrease from basal MEVA (*P* < 0.01).

There was an effect of time (*P* < 0.05) on the MEVA of both uterine horns, but this parameter was not affected by physiological status of the female (pregnant vs. non-pregnant; *P* = 0.9), laterality of ovulation (*P* = 0.4), nor by interactions between any of the variables measured. In pregnant and non-pregnant llamas with left-ovary ovulations the mean MEVA of right uterine horn displayed a significant (*P* < 0.05) decrease, compared to basal value, on day 10. On the contrary, in non-pregnant llamas with right-ovary ovulations the MEVA of the left uterine horn displayed a significant (*P* < 0.05) increase on day 20. The mean MEVA for both uterine horns, in pregnant and non-pregnant llamas with right or left ovary ovulations, during the entire period of evaluation are shown separately in Figure 2.

## Discussion

In the present study regardless of laterality of ovulation, intrauterine embryo location did not induce changes in mesometrial/endometrial vascularization area between the right and left uterine horn, during the phases of embryo migration, elongation and implantation in llamas.

The measurement of MEVA has been reported to be a reliable and sensitive tool to evaluate uterine blood flow during early gestation in mares and heifers (11, 16, 17). Also, using this ultrasonographic method our research group has demonstrated in previous studies (23, 24) that significant changes in uterine blood flow and vascularization area occur in llamas during the follicular growth phase or after mating. However, in the present study MEVA was similar for pregnant and non-pregnant females and between right and left uterine horns during the evaluation period.

A macroscopic anatomical study of uterine vascularization in llamas (4) has described the presence of a peculiar arrangement involving a prominent cross-over arterial branch extending from the right uterine artery to the left uterine horn, which could suggest that the left uterine horn is irrigated with a greater blood flow. However, the results of the present study were not able to detect a differential vascularization between uterine horns, regardless of the of female's physiological status or laterality of ovulation, therefore, not supporting the previously cited study (4).

Moreover, a significant individual variation regarding basal MEVA (i.e., pre mating) was observed among female llamas; however, no trend favoring a greater vascularization toward the left uterine horn was established. This great individual variation in uterine hemodynamic parameters has been reported by other studies in mares and it was not related to the stage of the cycle, age or parity (18, 20). Although several studies have described the hemodynamic changes during gestation in a variety of farm animal species, only a few (11, 16, 17) were conducted to evaluate hemodynamic changes during the embryonic peri implantation period.

In mares and cows (10, 20) the establishment of pregnancy gradually increases uterine arterial blood flow in accordance with embryo/fetal growth during the entire length of gestation period. These modifications seem to begin during the pre-implantation phase of embryo development (11, 16, 17) and exponentially increase thereafter (10). Interestingly, these modifications in uterine and endometrial vascular irrigation already begin during the histotrophic phase of embryo nutrition (17), when there is no intimate contact between the embryo and the endometrium, and are closely related to embryo location (11, 16, 17).

In the bovine, a species that does not present intrauterine embryo migration (i.e., the embryo remains in the uterine horn ipsilateral to the ovary from which ovulation occurred) compared to the equine, there is a clear increase in blood flow in the uterine artery ipsilateral to the uterine horn containing the embryo during the first weeks of gestation (11, 25). In heifers, the increase in uterine blood flow is directly correlated to subtler changes in endometrial vascular perfusion, and it begins as early as Day 13 (25) or 18 (11) of pregnancy. This last study demonstrated a temporal synchrony between the increase in uterine/endometrial vascular irrigation and embryo elongation, which in turn is closely related to the beginning of adhesiveness of the chorion to the endometrium (Day 20; 11), suggesting that the direct contact of the embryo with the endometrium induces local changes in uterine/endometrial blood flow.

On the contrary, in mares in which pre-implantation embryo develops an intense intrauterine migration before fixation occurs (26), endometrial vascular irrigation increased in an alternate manner between uterine horns, which was tightly synchronized with embryo location. Accordingly, during the period of intense intrauterine migration even the presence of the embryo for periods of 7 min, or longer in one location, determined a localized increase in endometrial vascular perfusion (16); thus, during the pre-implantation phase embryo-induced changes in endometrial vasculature parallel embryo migration between uterine horns (16, 17). However, shortly after embryo fixation the increase in endometrial blood flow was only observed in the endometrium surrounding the fixed embryonic vesicle (16). Moreover, from fixation day onwards, the blood flow of the uterine artery ipsilateral to the horn containing the embryo increased drastically compared to its contralateral counterpart (20).

However, in the present study the MEVA was similar in all the categories evaluated. There was no change in uterine vascularization between uterine horns in pregnant and non-pregnant females or between those whose embryos were originated from left- or right-sided ovulations. Furthermore, MEVA did not increase significantly over time during the first 30 days of gestation in the pregnant group as was described for the bovine, where it increased from Day 13 or 18 of pregnancy (11, 25). The reasons for these differences with observations made in other species could be due to a slower rate of llama embryo/fetus development during the first 3 months of gestation, as measured by crown-rump length, compared to cattle, sheep and horses (27, 28).

The vast differences in uterine and endometrial vascular irrigation during the early phase of embryo development, between species that display different embryonic strategies to signal its presence to the dam, could be related to the secretion of vascular stimulants into the uterine lumen by the embryo (17). In this regard, several studies have demonstrated that the Day 16 bovine embryo (29), and specially the equine embryo, as early as Day 12 (30, 33) produce and secrete estrogen, a molecule involved in inducing uterine contractility (26) and significant increases in uterine blood flow (31). Thus, during pre-implantation embryo development, in the bovine this molecule would be secreted into just one uterine horn, while in the mare it would be evenly distributed between both horns and the uterine body, inducing the previously described vascular changes. Despite the fact that estradiol has also been suggested as the most probable signaling candidate responsible for maternal recognition of pregnancy and intrauterine migration for the llama blastocyst (32), our results do not show an effect of embryo signaling on uterine blood flow. Larger quantities of estradiol secreted by the equine blastocyst compared to the llama embryo (32, 33), could explain the described effect in mare uterine blood flow and the absence of it in llamas.

Although in the present study embryo location did not induce changes in MEVA between the right and left uterine horn during the first month of gestation in llamas, there was an effect of time on uterine horn blood flow. Considering the slower rate of development of llama embryo/fetus during the first months of gestation, future investigations should consider a longer observational period to determine potential interactions between embryo and uterine blood flow and should increase the number of animals per group.

Finally, similar to our results, Travassos-Beltrame et al. (13) did not find differences between hemodynamic parameters between left and right uterine horns in pregnant sheep. Even though they started the Doppler ultrasonographic evaluation after pregnancy diagnosis was made on Day 28, hemodynamic variables were not affected by uterine horn nor single vs. multiple gestations.

Contrary to expectations, based on our results we can conclude that regardless of laterality of ovulation, in pregnant llamas the left horn did not display a greater MEVA before or after embryo arrival, a trend that was observed during the first 30 days of gestation.

## Data Availability Statement

The raw data supporting the conclusions of this article will be made available by the authors, without undue reservation.

## Ethics Statement

The animal study was reviewed and approved by Universidad Católica de Temuco.

## Author Contributions

MS and MR designed the experiment and wrote the manuscript. MS and FU developed the field work and analyzed the data. All authors contributed to the article and approved the submitted version.

## Conflict of Interest

The authors declare that the research was conducted in the absence of any commercial or financial relationships that could be construed as a potential conflict of interest.
